# Editorial: Clinical management of cholangiocarcinoma: surgical approaches and therapies

**DOI:** 10.3389/fonc.2024.1456958

**Published:** 2024-07-29

**Authors:** Hideo Takahashi, Noah A. Cohen, Kazunari Sasaki, Ganesh Gunasekaran

**Affiliations:** ^1^ Department of Surgery, Icahn School of Medicine at Mount Sinai, New York, NY, United States; ^2^ Division of Abdominal Transplant, Department of Surgery, Stanford University, Palo Alto, CA, United States

**Keywords:** cholangiocarcinoma, clinical management, editorial, review, hepato-pancreatobiliary surgery

Surgical and overall management of cholangiocarcinomas (CCAs), including intrahepatic CCAs, perihilar CCAs, and distal CCAs, can vary significantly based on the tumor’s location (1, 2). With the rising global incidence of CCA (3), there is an urgent need for effective early diagnosis and treatment strategies to reduce the burden of this devastating disease. The current standard treatment for resectable CCA involves surgical resection followed by adjuvant capecitabine (4). However, only a small fraction of patients present with resectable disease (1). Furthermore, even after curative resection, recurrence and metastasis are common, necessitating a multidisciplinary approach for advanced cases, often involving systemic chemotherapy (5). Similar to other oncological fields (6), targeted therapy and immunotherapy, in conjunction with surgical resection, have been utilized in CCA management, but their use has not been established fully (7).

The goal of this Research Topic is to compile and summarize the expanding knowledge on the surgical and systemic management of CCAs, as well as explore future directions. This Research Topic, Clinical Management of CCA: Surgical Approaches and Therapies, includes five publications; one original research, one case report, and three systematic review articles.

Wei et al. conducted original research investigating the potential of combining hepatic arterial infusion chemotherapy (HAIC) with lenvatinib, with or without PD-1 inhibitors, to improve clinical outcomes in patients with advanced CCA. Their study enrolled 55 patients between 2019 and 2022, divided into two groups: 35 patients received HAIC plus lenvatinib with PD-1 inhibitors (HAIC+LEN+PD-1i), and 20 patients received HAIC plus lenvatinib (HAIC+LEN). The HAIC+LEN+PD-1i group demonstrated significantly longer progression-free survival (PFS) and overall survival (OS) compared to the HAIC+LEN group with median PFS of 6.5 vs. 3.5 months (Hazard ratio (HR)= 0.390; 95% CI 0.189- 0.806; p= 0.001 and median OS of 16 vs. 11 months (HR= 0.461; 95% CI 0.229-0.927; p= 0.01)). The toxicities between two groups did not differ significantly. Despite being a small single-institution study, this study suggests a potentially effective treatment strategy for advanced CCA.

Li et al. presented a case report of the challenging diagnosis of IgG4 sclerosis cholangitis (IgG4-SC). A 55-year-old male patient presented with persistent jaundice and was initially diagnosed with perihilar cholangiocarcinoma. After surgical resection, pathological examination was positive for IgG4 plasma cells, leading to a diagnosis of isolated IgG4-SC. Although the patient initially responded well to steroid therapy, he became steroid-dependent and showed poor response to subsequent rituximab treatment. The case report underscores the importance of distinguishing IgG4-SC from cholangiocarcinoma to avoid unnecessary surgical interventions and highlights the need for better diagnostic tools and effective treatment strategies, particularly for patients who do not respond to steroids.

A systematic review and meta-analysis performed by Zhou et al. focused on the clinical outcomes of immune checkpoint inhibitor (ICI) combination therapy for advanced biliary tract cancer including CCAs. Analyzing 15 studies with 665 patients, they found that ICI combination therapy significantly improved the objective response rate, disease control rate, PFS, and OS compared to single ICI therapy or other treatments. However, they noted limitations such as study heterogeneity, lack of comparative studies, and publication bias. These findings highlight the need for further prospective randomized controlled trials to confirm the results and explore the effects of different ICIs combination regimens.

Wang et al. reviewed the clinical significance and critical role of small extracellular vesicles (sEVs), including exosomes and microparticles, in CCA. Their review outlined the involvement of sEVs in various biological processes that contribute to tumor cell proliferation, invasion, and metastasis within tumor microenvironment (TME). The authors emphasized that, due to their unique composition, stability, and accessibility in biological fluids, sEVs hold significant potential as biomarkers for early diagnosis and outcome prediction of CCA through liquid biopsies. Additionally, the properties of sEVs, such as specific tissue tropism and the ability to penetrate biological barriers, make them promising candidates for drug delivery systems in cancer therapy. The manuscript highlights the potential of sEV-based therapies for future clinical applications.

Lastly, Ruff and Pawlik reviewed various treatment options for intrahepatic CCA, including surgical and systemic treatments. They highlighted that surgical resection with clear margins is a cornerstone for improved outcomes in patients deemed resectable based on advanced imaging. Portal lymphadenectomy is recommended for staging, as lymph node metastases are associated with significantly worse outcomes. The debate between anatomic versus non-anatomic resection continues, but achieving an R0 resection appears more critical. The treatment of multifocal disease remains controversial, with some patients benefiting from surgical resection or hepatic artery infusion pumps (HAIP). Systemic therapies include cytotoxic chemotherapy, with gemcitabine and cisplatin as the cornerstone; recent studies suggest potential benefits of adding nab-paclitaxel to this regimen. Neoadjuvant chemotherapy is being explored for high-risk resectable CCA, while adjuvant capecitabine remains the standard after resection at this moment. Targeted therapies, particularly FGFR and IDH inhibitors, such as infigratinib, futibatinib, and pemigatinib, have shown promising outcomes in patients with specific tumor mutations. ICI is also under investigation for its potential role in the CCA treatment.

This editorial highlights recent advances in surgical and systemic management of CCA, and highlights future management directions. We hope that this Research Topic will guide multidisciplinary teams, including surgical oncologists, medical oncologists, radiation oncologists, and ancillary care teams, in managing this complex and devastating disease.

## Author contributions

HT: Writing – original draft, Writing – review & editing. NC: Writing – review & editing. KS: Writing – review & editing. GG: Writing – review & editing.
